# SMS-Based Active Surveillance of Adverse Events following Immunization in Children: The VigiVax Study

**DOI:** 10.3390/vaccines12091076

**Published:** 2024-09-20

**Authors:** Laura Augusta Gonella, Francesca Moretti, Annalisa Capuano, Caterina De Sarro, Lorenza Ferrara, Elisabetta Geninatti, Greta Guarnieri, Xhikjana Hysolakoj, Margherita Lalli, Olivia Leoni, Antea Maria Pia Mangano, Patrizia Marani Toro, Viviana Mecchia, Maria Caterina Merlano, Caterina Palleria, Anna Maria Potenza, Paola Rossi, Marco Rossi, Francesca Sanità, Ester Sapigni, Cristina Scavone, Claudia Sommaro, Marco Tuccori, Giovanna Zanoni, Ugo Moretti, VigiVax Working Group

**Affiliations:** 1 Section of Pharmacology, Department of Diagnostics and Public Health, University of Verona, 37134 Verona, Italy; lauraaugusta.gonella@univr.it; 2 Section of Hygiene and Environmental Occupational Preventive Medicine, Department of Diagnostics and Public Health, University of Verona, Piazzale L.A. Scuro 10, 37134 Verona, Italy; francesca.moretti@univr.it; 3 Department of Experimental Medicine, University of Campania “Luigi Vanvitelli”, Via Costantinopoli 16, 80138 Napoli, Italy; annalisa.capuano@unicampania.it (A.C.); cristina.scavone@unicampania.it (C.S.); 4 Unit of Clinical Pharmacology and Pharmacovigilance, “Renato Dulbecco” University Hospital, Research Center FAS@UMG, Department of Health Science, Magna Graecia University, 88100 Catanzaro, Italy; catedesarro@gmail.com (C.D.S.); palleria@unicz.it (C.P.); 5 Local Unit Health of Asti, Via Conte Verde 125, 14100 Asti, Italy; lferrara@asl.at.it; 6 Regional Center of Pharmacovigilance, Piemonte Region, Via Silvio Pellico 19, 10125 Torino, Italy; elisabetta.geninatti@aslcittaditorino.it; 7 Unit of Pharmacovigilance & Clinical Research, ASST Fatebenefratelli-Sacco, Department of Biomedical and Clinical Sciences, Università degli Studi di Milano, 20157 Milan, Italy; guarnieri.greta@asst-fbf-sacco.it; 8 Regional Center of Pharmacovigilance—Friuli Venezia Giulia Region, Department of Central Health, Social and Disability Policies, Via Cassa di Risparmio 10, 34100 Trieste, Italy; xhikjana.hysolakoj@regione.fvg.it (X.H.); viviana.mecchia@regione.fvg.it (V.M.); paola.rossi@regione.fvg.it (P.R.); claudia.sommaro@asfo.sanita.fvg.it (C.S.); 9 U.O.C. Farmaceutica Territoriale, Azienda Sanitaria Territoriale di Macerata, Belvedere Raffaello Sanzio 1, 62100 Macerata, Italy; margherita.lalli@sanita.marche.it; 10 Lombardy Regional Centre of Pharmacovigilance and Regional Epidemiologic Observatory, Welfare General Directorate, Lombardy Region, Piazza Città di Lombardia 1, 20124 Milan, Italy; olivia_leoni@regione.lombardia.it; 11 Regional Center of Pharmacovigilance, Marche Region, Via Gentile da Fabriano, 60125 Ancona, Italy; anteamp.mangano@regione.marche.it; 12 Health Office, Epidemiology and Public Health, ASL Pescara, Regional Department of Prevention Abruzzo, Via R. Paolini, 47, 65100 Pescara, Italy; patrizia.maranitoro@asl.pe.it; 13 ALISA/Liguria Region, Via Gabriele D Annunzio 64, 16121 Genova, Italy; caterina.merlano@alisa.liguria.it; 14 Regional Center for Pharmacovigilance, Emilia-Romagna Region, Medicines and Medical Devices Governance Area, Hospital Care Sector, General Directorate for Personal Care, Health and Welfare, Viale Aldo Moro 21, 40127 Bologna, Italy; annamaria.potenza@regione.emilia-romagna.it (A.M.P.); ester.sapigni@regione.emilia-romagna.it (E.S.); 15 Department of Medical, Surgical and Neuroscience Sciences, University of Siena, Viale Mario Bracci 16, 53100 Siena, Italy; marco.rossi@unisi.it; 16 Territorial Assistance Service, ASL Pescara, Regional Center of Pharmacovigilance, Abruzzo, Via R. Paolini 47, 65100 Pescara, Italy; francesca.sanita@asl.pe.it; 17 Unit of Adverse Drug Reaction Monitoring, University Hospital of Pisa, Via Roma 55, 56126 Pisa, Italy; marco.tuccori@gmail.com; 18 Immunology Unit, Pathology and Diagnostics Department, University Hospital of Verona, 37134 Verona, Italy; giovanna.zanoni@univr.it

**Keywords:** active vaccine safety surveillance, adverse event following immunization, short message services, pediatric vaccination, patient reporting, immunization registries, digital technologies, digital innovation for vaccine safety surveillance, serious adverse events

## Abstract

Underreporting is the main limitation of spontaneous reporting systems. This cohort-event monitoring study aims to examine the potential of short message service (SMS)-based surveillance compared to traditional surveillance systems. Using VigiVax software, parents of vaccinated children aged two years or younger, in the period March 2021–May 2022, received a single SMS inquiry about adverse events following immunization (AEFI). Responses were collected, validated by health operators and integrated with the information on electronic immunization registries. AEFI reports were automatically submitted to the Italian Pharmacovigilance system. Among 254,160 SMS messages sent, corresponding to 451,656 administered doses (AD), 71,643 responses were collected (28.2% response rate), and 21,231 of them (8.3%) reported AEFI. After a seriousness assessment based on clinical criteria, 50 reports (0.24%) were classified as serious. Among these, a causality assessment identified 31 reports at least potentially related to the vaccination (RR: 6.86/100,000 AD). Febrile seizures following MMRV (measles, mumps, rubella, varicella) vaccination accounted for 11 of these 31 cases, with an incidence of 32 per 100,000 AD. No fatal outcomes were reported. Our findings support the highly favorable risk profile of pediatric vaccinations and the possibility to improve spontaneous reporting through the integration of digital technologies.

## 1. Introduction

The latest change in European Union (EU) pharmacovigilance legislation (Regulation (EU) No 1027/2012 and Directive 2012/26/EU 2012) highlighted the importance of strengthening public participation in drug and vaccine safety monitoring [1]. Indeed, the legislation introduced the opportunity and right for European citizens to report any suspected adverse events related to drugs or vaccines (i.e., Adverse Events Following Immunization—AEFI) directly to national medicine regulatory authorities and marketing-authorization holders [2,3]. Patient engagement in vaccinovigilance activities offers several advantages, such as ensuring more timely detection of potential unknown threats, enhancing the system’s reliability through a more comprehensive understanding of vaccine safety, and fostering public confidence in regulatory authorities and vaccines [4,5]. Concerns about vaccine safety and potential adverse reactions are primary determinants of vaccine hesitancy. Establishing open and transparent communication regarding safety issues raised by the public can contribute to bolstering confidence in vaccination and enhancing adherence rates [6].

However, underreporting and low patient participation in post-marketing surveillance are well-known limitations of passive surveillance systems, potentially undermining the validity of collected data and negatively impacting benefit/risk assessment evaluations [7,8]. According to a recent study, the reporting rate (RR) for AEFI in Italy over a 10-year period (2008–2017) was 17.1 per 100,000 distributed doses [9]. The analysis showed a progressive increase over time, with notable peaks attributed to the implementation of active surveillance projects. However, the overall proportion of reports from citizens was low (4.4%), with nearly half collected in 2017, suggesting the need for the further promotion of citizen engagement in vaccinovigilance activities.

In a recent systematic review, the lack of competence in identifying/recognizing adverse reactions, lack of knowledge regarding the reporting process, as well as time constraints have been identified as the motivations or obstacles influencing patient reporting. Furthermore, another contributing factor identified was patient-healthcare provider communication issues, highlighting instances where Healthcare Professionals (HCP) may not adequately inform patients about reporting procedures or may even discourage patient reporting [10]. Simplifying the reporting process and promoting citizens’ awareness of their potentially valuable contribution to drug safety monitoring are essential steps to increase patient engagement. Various digital solutions, such as the utilization of short message service (SMS), have been implemented in recent years to enhance the quantity and quality of spontaneous reporting, facilitating active patient-centered surveillance projects and complementing passive surveillance data [11,12,13]. Moreover, many recent projects have been developed to apply Artificial Intelligence (AI), machine learning and Natural Language Processing (NLP) not only in pharmacovigilance data collection but also in the processing, identification and analysis of safety-relevant data [14,15]. Despite the promising nature of these systems in facilitating vaccine safety surveillance, they face challenges in ensuring the easy and feasible collection of all necessary data for both patients and HCPs. Balancing the need for comprehensive data collection by minimizing the burden on operators and ensuring user-friendly interfaces is crucial for maximizing participation and avoiding dropouts from surveillance.

The main aim of this study is to examine the potential of SMS-based surveillance of AEFI by integrating the use of SMS to prompt citizens’ reporting with data from electronic immunization registries (IRs) available in Local Health Units (LHUs) across Italian regions. The secondary aim is to provide safety surveillance data on consistent serious adverse reactions following pediatric vaccinations.

## 2. Materials and Methods

The Italian Pharmacovigilance system encompasses both drugs and vaccine surveillance; reports of AEFI are submitted to the Local Responsible for Pharmacovigilance (LRPV) and collected in the National Pharmacovigilance Network (NPN), managed by the Italian Medicines Agency (Agenzia Italiana del Farmaco—AIFA). This cohort-event monitoring study was coordinated by the Regional Pharmacovigilance Center (RPVC) of the Veneto Region of Italy in collaboration with AIFA. Eleven Italian regions, each with a computerized IRs, agreed to take part in the project (Table S1). The number of participating vaccination centers varied among regions, based on voluntary participation. All parents of children aged 2 years or younger who received compulsory and/or recommended vaccinations according to the regional vaccination plan aligned with the Preventive National Vaccination Plan (PNVP 2017–2019) [16], at one of the participating centers between 1 March 2021 and 31 May 2022, were considered eligible to participate in the study. A specific software, named ‘VigiVax’, was developed by the RPVC of the Veneto Region (Version 1.0). Personal data regarding patient initials, sex, date of birth, vaccine administration date, type of vaccine and mobile phone number of parents were extracted from the IRs and automatically uploaded on a weekly basis in VigiVax. Further steps consisted of the automatic generation and sending of one SMS to the parents of vaccinated children, asking whether any AEFI had occurred. The parents’ responses sent to the system by SMS were automatically integrated by VigiVax with the previously uploaded individual data. The software also included an NLP algorithm to prioritize the evaluation of SMS with any potential serious events. Sensitive data, such as phone numbers, were automatically deleted 30 days after the SMS was sent; however, parents still had the opportunity to respond within a reasonable timeframe, regarding the occurrence of any adverse reactions.

**Figure 1:**
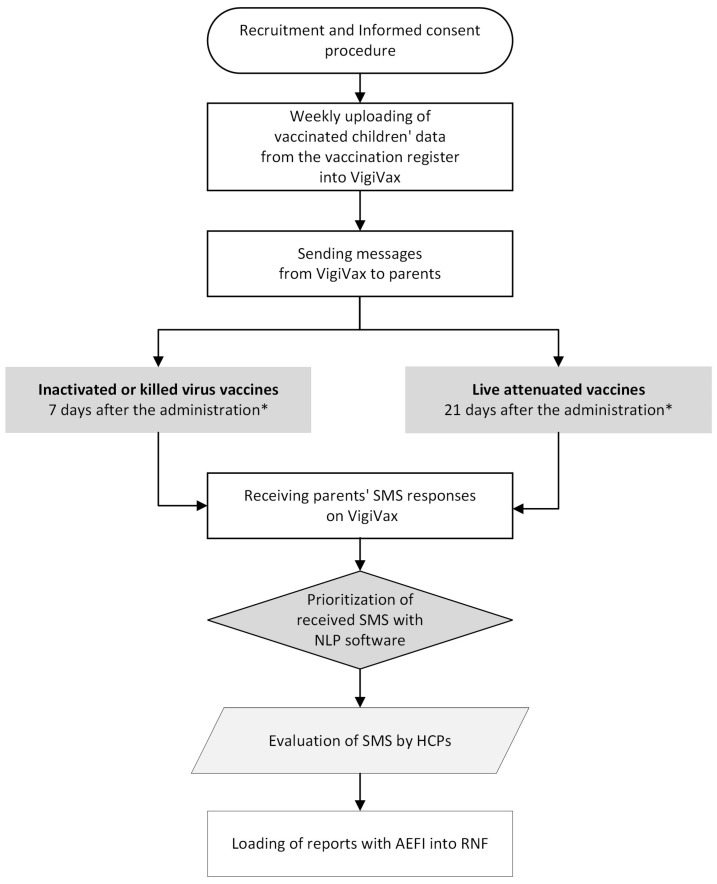
Flow chart of main steps of the project * When live and inactivated vaccines were co-administered in the same vaccination session, the interval for SMS sending was 21 days.

The data collection procedure involved several steps (Figure 1). Initially, all participating vaccination centers registered on VigiVax, and HCPs underwent training on software usage through video tutorials, online learning sessions, and instruction manuals. Subsequently, HCPs were tasked with recruiting all eligible patients during routine vaccination sessions, informing them about the study’s main purpose and data collection methods. Parents willing to participate in the project agreed to receive the SMS. In our study, less than 0.2% of parents declined participation. The SMSs were sent 7 or 21 days after vaccination, with inactivated or live attenuated vaccines, respectively. If a child received both a live and an inactivated vaccine, the message was sent after 21 days. Supplementary Table S2 specifies the types of vaccines and the timing for the SMS delivery. The SMS text inquired whether an AEFI had occurred after the vaccine administration and eventually asked to describe symptoms and their onset date.

The SMS responses from parents were collected in VigiVax without any time limits and were reviewed by expert HCPs at the vaccination center who previously assessed the seriousness of the reported AEFI. Based on clinical assessments, the vaccination center could request additional follow-up information for serious cases according to the routine activity. SMS responses without any reported AEFI were archived. Furthermore, SMS responses reporting any AEFI, that were evaluated by HCPs and deemed by the coordinating center as definitely unrelated to vaccine administration due to an incompatible time of onset or a definite relationship with other causes, were discarded. Finally, all SMS responses reporting AEFI, at least potentially related to vaccination, were entered into the NPN.

The reported adverse reactions were coded using the Medical Dictionary for Regulatory Activities (MedDRA) while vaccines were categorized based on national terminology and classification.

Reports were sent and classified according to seriousness in the pharmacovigilance system, starting from the judgement of the reporter, based on standardized criteria. Serious adverse reactions are defined as “any untoward medical occurrence that at any dose results in death, is life-threatening, requires inpatient hospitalization or prolongation of existing hospitalization, results in persistent or significant disability or incapacity, or is a congenital anomaly/birth defect” [17]. Moreover, reporters can classify an event as serious if they judge it to be clinically relevant. However, this approach may not always align with the actual clinical severity of the event [18]. All received reports were subsequently reevaluated, and their seriousness was reclassified by the coordinating center personnel based on clinical severity criteria. These criteria include cases displaying clinical manifestation requiring prolonged medical treatment, necessary hospital admission, cases with neurological complications or with permanent sequelae, congenital anomalies, neonatal pathologies, cases involving life-threatening situations or fatal outcomes [19]. The coordinating center also assessed the causal relationship between AEFI and the suspected vaccine for all the reports defined as serious by the reporters or during the revaluation. This assessment involves estimating the probability that the reported event may or may not be causally related to the vaccination and is carried out based on the WHO standardized algorithm [20]. The evaluation of the causal relationship considered factors such as temporal interval, presence of alternative causes, biological plausibility, epidemiological data, diagnostic investigations, therapy, duration, and clinical progression. The final classification includes the categories ‘consistent’ (when a causal association to immunization is established), ‘indeterminate’ (when the relationship cannot be definitively established or excluded), ‘inconsistent’ (when the causal relationship is excluded), or ‘unclassifiable’ (when there is insufficient data to determine the causal relationship).

The overall RR per administered doses (AD) was calculated as the number of reports submitted online per 100,000 AD. Based on the causality assessment, the RR for severe consistent and indeterminate AEFI per 100,000 AD was provided.

## 3. Results

During the study, 254,160 SMS messages were sent, for a total of 451,656 vaccine AD. There was significant variability among participating regions according to the number of involved vaccinations centers, with 66% of SMS messages sent from the Piemonte region and 20% from the Friuli Venezia Giulia region (Table S1). In terms of sex distribution, 51.3% of SMS messages referred to vaccinated male children.

**Figure 2:**
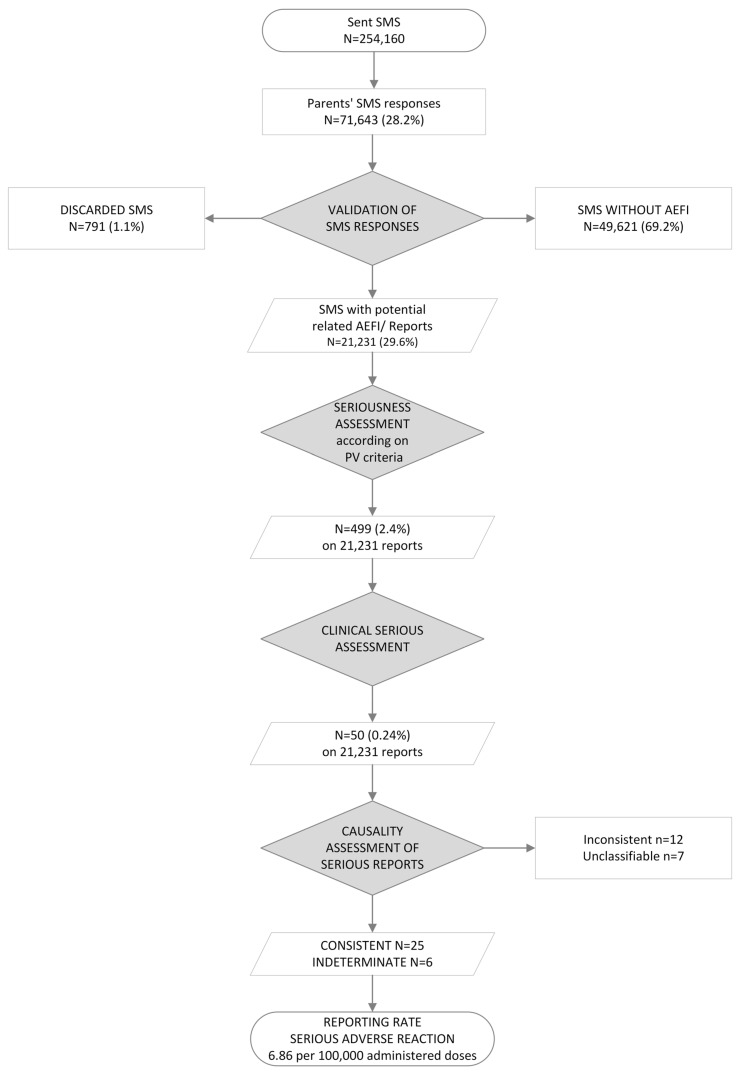
Flowchart illustrating the steps and results of the study.

An overall response rate of 28.2% was achieved (number of returned SMS messages = 71,643), with a distribution of 28.8% for males and 27.6% for females. Among all received responses 1.1% (791/71,643) of SMS messages were discarded after evaluation by the HCP. Furthermore, 69.3% (49,621/71,643) of responses affirmed the absence of any adverse event. Finally, 29.6% (21,231/71,643) reported at least one AEFI equivalent to 8.3% of the total number of sent SMS messages. Figure 2 shows steps and results of the study.

Considering sex distribution, the proportions of SMS responses were 29.8% for males and 29.3% for females (M/F RR Ratio 1.07, 95% CI 1.04–1.10), equivalent to 8.58% for males and 8.08% for females of sent SMS responses.

Vaccines administered in each vaccination session with a number of doses higher than 200 are listed in Table 1.

The 20 most frequently reported AEFI among the 21,231 notifications are shown in Table 2.

All cases reviewed and submitted by the HCPs were evaluated as serious according to pharmacovigilance criteria, with 499 out of 21,231 reports (2.35%) classified as ‘serious’. These mostly referred to clinically significant events but generally resolved spontaneously. The majority of these events included hyperpyrexia (54%), a combination of non-serious events (33%) or reactions that resulted in unnecessary emergency department visits. According to the seriousness assessment based on actual clinical criteria, 50 reports (0.24% of SMS responses with AEFI) were evaluated as serious, corresponding to an RR per 100,000 AD of 11.07 (RR per 100,000 vaccination session = 19.67). The causality assessment was inconsistent in 12 reports, while in seven cases, despite the follow-up, it remained unclassifiable (Table 3).

Finally, a total of 31 reports were classified as either “consistent” (N = 25, RR per 100,000 AD = 5.54) or indeterminate (N = 6, RR per 100,000 AD = 1.33), accounting for a proportion of 0.15% among all received SMS responses with AEFI and an overall RR per 100,000 AD of 6.86 (RR per 100,000 vaccination session = 12.2) (Table 4). Considering sex distribution, the incidence proportion of consistent/indeterminate reports was 0.014% for males and 0.011% for females of sent SMS responses (M:F RR Ratio 1.31, 95% CI 0.64–2.68).

The majority of serious AEFI refer to febrile seizures following MMRV vaccinations (n = 11), with an incidence of 32.35 per 100,000 doses (11/34,008). Of these reported cases, 73% were male (8 cases for males compared to 3 cases for females) with a RR Ratio M:F of 2.53 (95% CI 0.67–9.53). The RR for specific vaccines for other notable adverse events include 3.93 cases per 100,000 doses of febrile seizures after the MenB vaccine (4/101,829), 3.17 cases per 100,000 doses of intussusception after the rotavirus vaccine (2/63,028), 2.79 cases per 100,000 doses of thrombocytopenia after the MMRV and MenC-con vaccine (1/35,884) and 0.98 cases per 100,000 doses of thrombocytopenia after the MenB vaccine (1/101,829).

Regarding the outcome of reported AEFI, 77.4% (24 out of 31 cases) resulted in complete recovery or improvement, while data for the remaining cases weres either not available at follow-up or were unknown at time of follow up. No fatal outcomes were reported.

## 4. Discussion

This study, conducted on a large sample of vaccinated children, confirms the excellent safety profile of vaccines and underscores the importance of integrating digital tools into vaccine surveillance.

Digital technology and mobile systems to enhance post-marketing surveillance of vaccines and drugs has been widely implemented in many countries. A recent scoping review, including twenty-seven publications, focused on the effectiveness of interventions like SMS, emails, etc. to enhance public participation in AEFI surveillance [11]. Most of the studies came from Australia and Canada, with the seasonal influenza vaccine being the most frequently monitored. Considering only publications comparable to our study (i.e., studies covering all vaccines scheduled for the pediatric population), we observed a notably lower response rate (28%), in contrast to response rates ranging from 70% to 90% in other studies [21,22]. However, it’s important to note that response rates are usually calculated based on the initial contact with vaccinees. Indeed, digital AEFI surveillance systems typically rely on an initial SMS or e-mail to inquire about the occurrence of an AEFI and if a positive answer is received, more specific information is gathered through subsequent e-questionnaires. In our study, both the occurrence of AEFI and all necessary information were collected via a single SMS contact, thanks to the integration with data from the IR. Actually, when considering the proportion of responses reporting at least one AEFI, our study obtained similar or even higher proportions. Specifically, Gold et al. [22] conducted a randomized controlled trial (RCT) to assess the efficacy of the Stimulated Telephone Assisted Rapid Safety Surveillance (STARSS) System. This initiative utilized repetitive SMS messages sent to adult vaccinees or parents of children receiving a vaccine and obtained an AEFI detection rate of 4.3%. In our study, the proportion of AEFI reports per total SMS messages sent was approximately two-fold higher (8.3%). Similarly, a study focused on the implementation of a vaccine safety monitoring tool based on SMS (SmartVax), to prompt AEFI reporting after childhood vaccination (<5 years), obtained a proportion of AEFI reports similar to our study (i.e., 8.2%; 239 out of 2897 vaccination visits) over a 44-month period [21].

It is important to consider that our study is temporally positioned during the pandemic period and throughout the entire COVID-19 vaccination campaign, which was characterized by a decrease in AEFI reporting for no-COVID-19 vaccine [23,24]. Indeed, HCPs and the general population primarily focused their efforts on the global emergency and on monitoring the COVID-19 vaccine rather than the more established childhood vaccines analyzed in our project. Additionally, the increased workload brought by the pandemic may have adversely affected the time dedicated to the project by HCPs, potentially resulting in less effort put into promoting the project to parents. To confirm the negative impact of the pandemic on vaccine surveillance not related to COVID-19, an RCTconducted around the same time as our research aimed at testing vaccine safety surveillance through SMS and computer-assisted telephone interviews (i.e., the Zimbabwe Stimulated Telephone Assisted Rapid Safety Surveillance—Zm-STARSS), achieved a response rate of 31% (704 out of 2280), comparable to our study. These findings confirm that VigiVax represents an efficient and effective method for enhancing the surveillance of vaccine adverse events [25].

Although the pandemic may have negatively affected the participation, it is worth noticing that the national AEFI RR of non-COVID-19 vaccines per 100,000 AD was higher in the year 2021 during the project’s occurrence (i.e., 78 per 100,000 AD) compared to spontaneous reporting in the previous years (17.9 and 22 per 100,000 AD in 2020 and 2019, respectively) [24,26]. These results suggest that the implementation of feasible active surveillance of AEFI ensured the maintenance of an adequate level of safety monitoring during the pandemic when the spontaneous reporting system experienced a significant decline, both in the context of drugs and vaccines.

One of our study’s strengths was its integration with information from regional IRs. This simplified parents’ participation by requiring responses via a single SMS and reduced the workload for HCPs, who no longer needed to collect patient data. In fact, data from the IRs, combined with AEFI descriptions provided by parents via SMS, allowed for the automatic generation of reports submitted to the NPN.

The timing for sending SMS messages was also adapted to the type of vaccine, differentiating between live and inactivated vaccines, which enabled a higher likelihood of capturing AEFI with respective timeframes of 21 and 7 days. For comparison, other similar active surveillance systems were designed to receive responses within a few hours after vaccination, potentially leading to the loss of relevant data [21]. It is noteworthy that in our system, parents had the opportunity to send SMS messages even outside the 7 or 21-day window with any further information automatically collated into the same report. Moreover, the minimal rate of discarded messages (1.1%) by HCPs indicates the reliability and credibility of the patient-reported data, which are crucial in the context of vaccine surveillance where there is a risk of receiving numerous reports of unrelated adverse events. This underscores the importance of involving patients in the adverse events reporting system, as they often provide insights into adverse reactions that may differ from those reported by HCPs. Furthermore, this highlights the crucial role of easily accessible surveillance systems that can facilitate public participation [27].

Additionally, the proportion of SMS messages reporting at least one AEFI was highly relevant, confirming the system’s usefulness in collecting valuable information for vaccine safety monitoring. It is important to note that all discharged SMS messages were confirmed by the coordinating center to avoid loss of pertinent information.

Finally, the population sample recruited for our project was notably larger than in other published initiatives for active surveillance based on SMS [11]. This substantial volume allowed us to thoroughly test the effectiveness of such a system, demonstrating its potential for routine implementation. Notably, a high proportion of responses was received even when no AEFI occurred. A separate analysis of these responses revealed that participating parents highly valued the system.

The most frequently reported MedDRA PTs such as fever, irritability, and pain at the injection site, are well-known and considered mild vaccine reactions for the pediatric population. Data presented in Table 1 suggests that vaccines administered during the same session are reported more frequently than those administered separately. For instance, the co-administration of MenB + PNEUMO-con13 and MenB + ROTAVIRUS shows greatly higher reporting rates compared to individual vaccines such as MenB and MMRV. However, the sum of individual RR for each type of vaccine administered separately is similar or even lower than the RR for the same vaccines administered during the same session, suggesting that there is no increase in risk when vaccines are co-administered compared to when they are administered separately. In an immunization schedule rich in co-administrations, active surveillance becomes crucial to ascertain whether there is a difference in risk associated with vaccine co-administration.

Borsari et al. [28] conducted a study comparing the agreement between Classification of AEFI Seriousness as defined according to pharmacovigilance standard criteria and Clinical Severity Classification used in an Italian region. The study revealed a low agreement of only 58% (Cohen’s Kappa = 0.17). To address this issue, the Green Channel, a specialized service for pre-vaccination counseling established in 1993 by the Veneto Region Public Health Authority, regularly re-evaluates reports of serious adverse events based on clinical severity criteria [29]. The re-classification of clinical severity of AEFI performed by experts plays a crucial role in improving public communication, providing accurate information about real serious risks, enhancing transparency, and contributing to the promotion of vaccinations [19].

In our study, the clinical evaluation of seriousness resulted in 50 confirmed serious cases, among which 31 had events at least potentially associated with vaccination, corresponding to an incidence of approximately 7 severe AEFI per 100,000 doses. These reports describe events known within the risk profile of the suspected vaccines. Furthermore, no fatal cases have been reported, and no sequelae have been found in the follow-up when available. The high number of collected reports, which underwent clinical reviews and severity assessments, increased the relevance of retrieved results, confirming the favorable risk profile of pediatric vaccinations.

In terms of sex distribution, a similar proportion of SMS messages was sent to both the male and female population (51% vs. 49%). However, when considering SMS reporting AEFI, male children showed a slightly higher significant risk of AEFI compared to females (with an increase between 3 and 9%). Moreover, male children showed a higher risk for serious events (approximately 30%), although this difference was not statistically significant. According to the literature, adult women have twice the risk of experiencing Adverse Drug Reactions (ADRs) compared to men, as well as a higher risk of hospitalization for ADRs [30]. However, a different trend has been observed in the pediatric population where male children seem to have a higher risk for serious or fatal events [31]. Furthermore, an analysis of ADR reports in the NPN on the relationship between age and sex among children indicates that males experience more adverse drug reaction episodes before the age of two years [32].

Our data are consistent with the available evidence. The retrieved higher risk for serious events in the male population appears to be particularly high for MMRV-induced febrile convulsions, with 73% of cases (among a limited number of 11 cases retrieved). While this difference is not statistically significant, the limited number of serious cases retrieved suggests the need for more extensive studies to better explore any disparities. Regarding the type of serious AEFI retrieved, the majority of reported cases were specifically associated with febrile seizures. As expected, according to the literature, most of these cases followed MMRV vaccinations [33,34] Despite being rare, febrile seizures are well-known events after MMRV vaccination; however, the literature shows a wide variability in their rate of occurrence, likely depending on several factors such as the methodology implemented for data collection, the duration of follow-up, the definition of the relationship with vaccinations, etc.

A recent systematic review estimated the incidence of febrile seizures after MMRV vaccination to be approximately 96 cases per 100,000 children during the second week post-vaccination, roughly three times higher than the rate found in our active surveillance project (32.3 per 100,000 doses) [35]. When comparing studies with age cohorts (i.e., under two years old) and follow-up periods similar to our study (i.e., within 21 days), the incident rates were nearly twice as high as ours: 58 per 100,000 doses occurring between 7–10 days after MMRV vaccination in a retrospective cohort study of a cohort aged 12–23 months, and 70 per 100,000 doses occurring between 5–12 days after MMRV in another retrospective cohort study of a cohort aged 12–60 months [36,37]. A systematic review and meta-analysis found the incidence rate in children aged 10–24 months ranging between 62 and 96 per 100,000, calculated for four retrospective cohort studies during the second week after vaccination. The same study found an incidence between 93 and 129 per 100,000 doses considering children aged 12–23 months 7–10 days after vaccination [38].

The observed discrepancy in febrile seizures rates between our project and those reported in the literature is probably due to the type of our study, which focuses on spontaneous reporting, even though stimulated. Febrile seizures are actually well-known reactions that generally do not result in serious consequences and resolve without any residual effects, as also observed in our data [39].

The literature demonstrates that the incidence of febrile seizures in the general pediatric population is significantly higher than the rate associated with vaccination (e.g., 4000 cases per 1,000,000 children) [35]. According to available data, the Centers for Disease Control and Prevention (CDC) highlights the crucial role of vaccines in preventing a greater number of febrile seizures cases by protecting children against diseases known to induce fever and febrile seizures (e.g., measles, mumps, rubella, chickenpox, influenza, pneumococcal infections, etc.) [40].

The observed rate of thrombocytopenia following the MMRV vaccine is consistent with data found in the literature. A recent Cochrane systematic review on the safety profile of all vaccines containing measles, rubella, mumps, and varicella strains in individuals under 12 years of age shows the incidence of Idiopathic Thrombocytopenic Purpura (ITP) at 1 case per 40,000 administered MMR doses compared to our result of 1 case of Thrombocytopenia per 34,008 after MMRV vaccination [41]. Cases of ITP following natural infection are higher (i.e., 5 cases per 100,000 individuals), thus confirming the favorable risk-benefit profile of vaccination.

Likewise, the retrieved rate of intussusception following the rotavirus vaccine (i.e., 3.17 per 100,000 doses) aligns with the available evidence. Based on CDC data, the risk within a week following the first or second dose is estimated to vary from 1 in 20,000 to 1 in 100,000 vaccinated infants who received the rotavirus vaccine in the US [42]. The background rate in Italy is estimated to be much higher, at 20 cases per 100,000 doses [43]. Two recent meta-analyses, one involving randomized controlled trials (with 104,647 participants receiving the vaccine and 95,947 receiving a placebo) and the other involving observational studies (with more than 4,500,000 participants receiving the first dose), examined the association between the rotavirus vaccine and intussusception [44,45]. The analyses found no associations in RCT and respectively a 3.7 increased risk for cohort studies and an 8.45 for case-control studies. An interrupted time series even demonstrated a decrease in intussusception incidence during the post-vaccine period compared to the pre-vaccine period [46]. Our data support the evidence that intussusception following rotavirus vaccine is a rare event that does not affect the risk-benefit ratio, which remains in favor of vaccination [47].

In conclusion, the results from our active surveillance project, based on a large pediatric population, confirm the favorable risk profile for childhood vaccines.

## 5. Strengths and Limitations

Regarding the strengths of our study, the results are based on a large cohort of the pediatric population and involve a systematic assessment of all collected reports to evaluate severity from a clinical perspective. This provides a reliable incidence rate of adverse events related or potentially related to vaccinations.

The main limitation of the study lies in the fact that, although only one initial contact was stipulated according to the protocol, systematic follow-up by HCPs to investigate any cases of non-serious adverse reactions was not guaranteed. However, follow-up is generally conducted in routine passive surveillance monitoring for serious events. Moreover, considering the positive acceptance from parents and HCPs of the VigiVax system, it could be easily systematically integrated in the future to allow the active collection of follow-up data.

## 6. Conclusions

These findings suggest that VigiVax is a valuable tool for vaccine safety assessment, especially when introducing new vaccine combinations or updates to the vaccination schedule. The integration of VigiVax with existing IRs and the adoption of a single SMS system for AEFI reporting have demonstrated promising results in terms of both effectiveness and efficiency, especially during the COVID-19 period. Moreover, the routine implementation of the NLP software will further enhance the efficiency and accuracy of HCPs’ pharmacovigilance activities by enabling fast recognition of all critical terms potentially indicating a serious event, thereby simplifying the analysis stage. The validation of the software will be the subject of a subsequent publication.

This approach not only ensures timely and accurate reporting of adverse events but also streamlines the surveillance process, contributing to the overall success of the surveillance program. Ensuring the long-term sustainability of VigiVax relies on maintaining this efficient workflow and minimizing the burden on HCPs, particularly amidst the ongoing demands of their regular duties. By optimizing these processes and reducing the workload of HCPs, we can ensure the continuity and effectiveness of VigiVax over time.

Implementing this methodology routinely is expected to increase the collection of adverse events with higher patient engagement in vaccinovigilance, contributing to a better understanding of vaccine safety and enabling more informed and secure clinical practices. Encouraging citizen reporting while maintaining system efficiency is paramount. Further innovative solutions are needed to efficiently foster surveillance systems and manage the workload of HCPs.

## Figures and Tables

**Table 1:** Number of reports and reporting rate for administered vaccines. Administered vaccines with higher Reporting Rates are highlighted in bold (MenB = Meningococcal B vaccine; DTaP-HB-IPV-HIB = Diphtheria, Tetanus, Pertussis, Hepatitis B, Polio, Haemophilus influenzae type b vaccine/Hexavalent vaccine; PNEUMO-con = Pneumococcal conjugate vaccine; ROTAVIRUS = Rotavirus vaccine; MENC-con = Meningococcal C conjugate vaccine; MMRV = Measles, Mumps, Rubella, Varicella vaccine; PNEUMO-con13 = 13-valent Pneumococcal conjugate vaccine; MenACWY = Meningococcal ACWY conjugate vaccine; INF = Influenza vaccine; VAR = Varicella vaccine; HA = Hepatitis A vaccine; MMR = Measles, Mumps, Rubella vaccine).

Administered Vaccines	N° Report	Administered Doses	Reporting Rate Per 10,000
MENB	9337	95,956	973.1
DTaP-HB-IPV-HIB + PNEUMO-con + ROTAVIRUS	2257	37,696	598.7
DTaP-HB-IPV-HIB + PNEUMO-con	2115	30,713	688.6
MENC-con + MMRV	1654	22,404	738.3
DTaP-HB-IPV-HIB + PNEUMO-con13 + ROTAVIRUS	1607	17,786	903.5
DTaP-HB-IPV-HIB + PNEUMO-con13	1454	15,138	960.5
**MenACWY + MMRV**	**798**	**7500**	**1064.0**
DTaP-HB-IPV-HIB	326	4912	663.7
**MenB + ROTAVIRUS**	**603**	**4579**	**1316.9**
MMRV	333	3492	953.6
ROTAVIRUS	84	1581	531.3
INF	30	1360	220.6
MenACWY	38	1019	372.9
DTaP-HB-IPV-HIB + ROTAVIRUS	49	857	571.8
VAR	26	835	311.4
PNEUMO-con	34	817	416.2
HA	5	751	66.6
MENC-con	16	722	221.6
PNEUMO-con13	33	622	530.5
MMR	36	420	857.1
**MenACWY + MMR**	**48**	**405**	**1185.2**
**MenB + PNEUMO-con13**	**56**	**371**	**1509.4**
HA + MENB	14	348	402.3
DTaP-HB-IPV-HIB + MenACWY	30	336	892.9
MENC-con + MMR	20	306	653.6
DTaP-HB-IPV-HIB + MMRV	16	284	563.4
**MenB + MENC-con**	**30**	**268**	**1119.4**

**Table 2:** Most reported AEFI on received reports (BT* = Body Temperature, ** in this term all adverse reactions at the vaccination site have been consolidated).

MedDRA PT (Preferred Term)	N° Reports	% on Reports
Fever (BT* ≤ 39.5 °C)	16,217	76.4%
Vaccination site reaction **	3440	16.2%
Irritability	1756	8.3%
Diarrhea	1039	4.9%
Restlessness	937	4.4%
Crying	871	4.1%
Nervousness	814	3.8%
Decreased appetite	775	3.7%
Skin rash	664	3.1%
Abdominal pain	564	2.7%
Somnolence	563	2.7%
Malaise	538	2.5%
Vomiting	493	2.3%
Rush morbilliform	393	1.9%
Fatigue	326	1.5%
Hyperpyrexia (BT* ≥ 39.5 °C)	282	1.3%
Agitation	259	1.2%
Sleep disorder	247	1.2%
Pain in extremity	219	1.0%
Constipation	213	1.0%

**Table 3:** Distribution of inconsistent and unclassifiable serious reports by main adverse reactions, administered vaccines and outcomes (* BT* ≥ 39.5 °C; MenB = Meningococcal B vaccine; MenACWY = Meningococcal ACWY conjugate vaccine; ROTAVIRUS = Rotavirus vaccine; DTaP-HB-IPV-HIB = Diphtheria, Tetanus, Pertussis, Hepatitis B, Polio, Haemophilus influenzae type b vaccine/Hexavalent vaccine; PNEUMO-con = Pneumococcal conjugate vaccine; MMRV = Measles, Mumps, Rubella, Varicella vaccine; MenC-con = Meningococcal C conjugate vaccine; MMR = Measles, Mumps, Rubella vaccine; VAR = Varicella vaccine.).

Main Adverse Reactions	N°	Administered Vaccine	Causality Assessment	Outcome at the Follow Up
Febrile Seizures	4	MenB (3); MenACWY (1 inconsistent)	Inconsistent (3); Unclassifiable (1)	Improvement (1); Not available/unknown (2); Complete Resolution (1)
Bronchiolitis	4	MenB + ROTAVIRUS; DTaP-HB-IPV-HIB + PNEUMO-con + ROTAVIRUS; MMRV + MenACWY; MenB	Inconsistent (4)	Complete Resolution (1); Not available/unknown (2); Improvement (1)
Bronchiolitis, otitis (Hospitalization)	1	MMRV + MenC-con	Unclassifiable	Not available/unknown
Periorbital cellulitis; MSSA (Methicillin-sensitive Staphylococcus aureus) ear infection; Perforation of the tympanic membrane	1	MMRV	Inconsistent	Complete Resolution
Trembling and cold sweat, after which the child fainted and momentarily stopped breathing (Hospitalization)	1	MenB	Unclassifiable	Not available/unknown
Convulsive Crisis	1	MMR	Unclassifiable	Not available/unknown
Epileptic seizure	1	MenB + ROTAVIRUS	Unclassifiable	Not available/unknown
Severe immunodeficiency (hospitalization and bone marrow transplant). A few hours after the vaccine, petechiae appeared.	1	MMRV	Inconsistent	Not resolved
Hyperpyrexia * (Hospitalization)	2	MenB (1); VAR (1)	Inconsistent (2)	Complete Resolution (1); Not available/unknown (1)
Hyperpyrexia *, severe vomiting and diarrhea (Hospitalization)	1	MMRV + MenC-con	Inconsistent	Complete Resolution
Generalized urticaria (hospitalization)	1	MenB	Unclassifiable	Complete Resolution
Thrombocytopenia	1	MMRV	Unclassifiable	Not available/unknown

**Table 4:** Distribution of consistent and indeterminate serious reports by main adverse reactions, suspected vaccines and outcomes (* BT* ≥ 39.5 °C, ** One of the cases of intussusception required surgical treatment. MMRV = Measles, Mumps, Rubella, Varicella vaccine; MenB = Meningococcal B vaccine; Hexavalent vaccine = Diphtheria, Tetanus, Pertussis, Hepatitis B, Polio, Haemophilus influenzae type b vaccine/Hexavalent vaccine; PNEUMO-con = Pneumococcal conjugate vaccine; PNEUMO-con13 = 13-valent Pneumococcal conjugate vaccine; MenACWY = Meningococcal ACWY conjugate vaccine; MenC-con = Meningococcal C conjugate vaccine; MMR = Measles, Mumps, Rubella vaccine; VAR = Varicella vaccine; ROTAVIRUS = Rotavirus vaccine.).

Main Adverse Reactions	N°	Suspected Vaccine	Causality Assessment	Outcome at the Follow Up
Febrile Seizures	21	MMRV (11 consistent); MenB (4 consistent); DTaP-HB-IPV-HIB + PNEUMO-con (1 consistent); DTaP-HB-IPV-HIB + PNEUMO-con13 (1 consistent); MenACWY (1 consistent); MenC-con (1 consistent); MMR (1 Indeterminate); VAR (1 Indeterminate)	Consistent (19); Indeterminate (2)	Complete Resolution (13); Improvement (3); Not available/unknown (5)
Intussusception **	2	ROTAVIRUS (2)	Consistent (1); Indeterminate (1)	Complete Resolution (2)
Thrombocytopenia	2	MMRV + MenC-con (1); MenB (1)	Indeterminate (2)	Complete Resolution (1); Not available/unknown (1)
Convulsive Crisis	1	DTaP-HB-IPV-HIB	Consistent	Complete Resolution
Hyperpyrexia * and severe vomiting (Hospitalization)	1	DTaP-HB-IPV-HIB + PNEUMO-con13 + ROTAVIRUS	Indeterminate	Complete Resolution
Hyperpyrexia * (Hospitalization)	1	MMRV	Consistent	Complete Resolution
Hyperpyrexia * (Hospitalization), Macular Rash	1	MMRV	Consistent	Not available/unknown
Petechiae on legs, arms, face (Hospitalization)	1	MMRV	Consistent	Complete Resolution
Allergic Reaction- Treated with steroids and antihistamine for macular rash and blisters. (Hospitalization)	1	MenB	Consistent	Complete Resolution

## Data Availability

The original contributions presented in the study are included in the article and Supplementary Material, further inquiries can be directed to the corresponding author.

## Supplementary Materials

The following supporting information can be downloaded at: https://www.mdpi.com/article/10.3390/vaccines12091076/s1, Table S1: Regional Distribution of Sent Messages and Parents’ Responses via SMS; Table S2: SMS Sending Timeframes for Various Vaccines.
