# Supplementary material for: SMS-Based Active Surveillance of Adverse Events following Immunization in Children: The VigiVax Study
Source: Vaccines (Basel). 2024 Sep 20;12(9):1076. doi: 10.3390/vaccines12091076 (PMC11435886; doi:10.3390/vaccines12091076)
Supplement: Supplementary file 1 [file vaccines-12-01076-s001.zip › vaccines-3161026-supplementary.pdf]

Table S1. Regional Distribution of Sent Messages and Parents' Responses via SMS. (* % on the total number of SMS sent; **% on the number of SMS sent by each region)

Region	Sent messages		Parents' Answers with SMS	
	N°	%*	N°	%**
**Abruzzo**	1,710	0.7	239	14.0
**Calabria**	1,738	0.7	242	13.9
**Campania**	2,933	1.2	992	33.8
**Emilia-Romagna**	10,152	4.0	3,881	38.2
**Friuli-Venezia Giulia**	50,913	20.0	14,811	29.1
**Liguria**	1,165	0.5	554	47.6
**Lombardia**	9,432	3.7	3,194	33.9
**Marche**	1,643	0.6	720	43.8
**Piemonte**	169,858	66.8	45,723	26.9
**Toscana**	1,869	0.7	343	18.4
**Veneto**	2,747	1.1	944	34.4
**Total**	254,160	100.0	71,643	28.2

Table S2. SMS Sending Timeframes for Various Vaccines.

Name of Vaccine	SMS sending time (days)
Cholera vaccine	7
Diphtheria, Tetanus vaccine	7
Diphtheria, Tetanus, Pertussis, Hepatitis B, Polio, Haemophilus influenzae type b vaccine (Hexavalent vaccine)	7
Diphtheria, Tetanus, Pertussis, Polio vaccine	7
Japanese encephalitis vaccine	7
Hepatitis A vaccine	7
Hepatitis B vaccine	7
Haemophilus influenzae type b vaccine	7
Influenza vaccine	7
Inactivated Polio Vaccine	7
Meningococcal ACWY conjugate vaccine	7
Meningococcal B vaccine	7
Meningococcal C conjugate vaccine	7
Measles, Mumps, Rubella vaccine	21
Measles, Mumps, Rubella, Varicella vaccine	21
Pneumococcal vaccine	7
Pneumococcal conjugate vaccine	7
13-valent Pneumococcal conjugate vaccine	7
Rabies vaccine	7
Rotavirus vaccine	21
Typhoid vaccine (injection)	7
Oral typhoid vaccine	21
Tetanus vaccine	7
Varicella vaccine	21
Yellow fever vaccine	21
Tick-borne encephalitis vaccine	7
